# Multiple memory formation in glassy landscapes

**DOI:** 10.1126/sciadv.abg7133

**Published:** 2021-08-11

**Authors:** Chloe W. Lindeman, Sidney R. Nagel

**Affiliations:** Department of Physics and The James Franck and Enrico Fermi Institutes, University of Chicago, Chicago, IL 60637, USA.

## Abstract

Cyclically sheared jammed packings form memories of the shear amplitude at which they were trained by falling into periodic orbits where each particle returns to the identical position in subsequent cycles. While simple models that treat clusters of rearranging particles as isolated two-state systems offer insight into this memory formation, they fail to account for the long training times and multiperiod orbits observed in simulated sheared packings. We show that adding interactions between rearranging clusters overcomes these deficiencies. In addition, interactions allow simultaneous encoding of multiple memories, which would not have been possible otherwise. These memories are different in an essential way from those found in other systems, such as multiple transient memories observed in sheared suspensions, and contain information about the strength of the interactions.

## Teaser

An interacting-rearrangement model for sheared solids shows multiple interaction-encoding memories and complex limit cycles.

## INTRODUCTION

Memories in matter can be created in a multitude of ways (*1*). Of interest here is a particular form of memory in which a jammed packing of particles subjected to training by a cyclic quasi-static shear can form a memory of the amplitude at which the shear was applied (*2*–*7*). The memory is encoded because the system falls into a periodic orbit in which the packing revisits precisely the same states during each applied shear cycle. The periodic orbit is disturbed if the shear amplitude is altered so that a memory of the training amplitude can be “read out” by tracking the particle displacements after cycles of increasing strain.

This memory formation is reminiscent of the ones found in non-Brownian suspensions (*8*–*12*). However, for such suspensions, each particle only interacts if it collides with a neighbor; in jammed particle packings, the particles are in enduring contact with their neighbors throughout each cycle. In this case, it is less clear how the memory is formed. The energy landscapes are different: The jammed systems have an exponential number of well-defined local energy minima, while the suspensions have very large flat-bottomed ground states. In the case of suspensions, the orbit is reversible along each cycle, and there are no energy barriers that need to be overcome. In contrast, jammed systems deform via rearrangements between clusters of particles that occur in one direction of the shear and that undo themselves at a different amplitude as the system is sheared in the reverse direction (*2*). The motion is thus not reversible within a cycle but is still periodic. Because jammed packings exist in a very rugged, high-dimensional, and complex energy landscape (*13*), it is astonishing that these systems can find a periodic orbit at all and, moreover, that the periodic orbit can be discovered relatively rapidly.

Aspects of the periodic memories encoded in jammed packings have been modeled in a variety of ways (*2*, *14*–*17*). We consider here a model that was motivated by the existence of localized regions in a disordered material, which are particularly prone to rearrangements due to applied external forcing (*14*). This model, on the basis of the Preisach model (*18*), originally proposed for magnetic systems, considers independent, noninteracting defects, each of which can exist in either of two states with an energy barrier between them. Because of an applied external strain, a defect can flip between the two states; however, the strain to flip in the “positive” direction is not necessarily the same as for it to flip in the opposite “negative” direction. Each defect, known as a hysteron, is thus an elementary unit of hysteresis in the system. While Preisach models have been successful at describing many aspects of the memories, there are certain phenomena that they do not capture at all.

Here, we generalize this type of model by including interactions between hysterons so that the applied strains for one hysteron to flip between its two states depends on the state of the others. As we will show, this generalization not only exhibits some of the phenomena not possible without interactions but also leads to a type of memory that, to our knowledge, has not yet been identified in other systems. Moreover, because hysteresis is embedded in the fundamental units that make up the model, we are able to go to the limit of very small system sizes and very small interaction strengths to isolate the effect of the interactions and the origin of these behaviors.

## RESULTS

### Noninteracting model—Successes and failures

Consider an ensemble of hysterons in the presence of an externally applied shear γ.

**Fig. 1:**
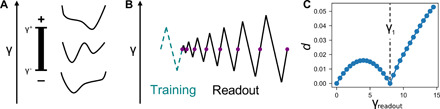
Hysteron schematic. (**A**) A single hysteron with flipping strains γ^+^ and γ^−^. The configuration can be modeled as a double-well potential as shown. For γ > γ^+^, the hysteron is forced into the (+) state (i.e., the rightmost well), and for γ < γ^−^, the hysteron is forced into the (−) state (i.e., the leftmost well). For intermediate values of the strain, γ^−^ < γ < γ^+^, the hysteron can reside in either state as prescribed by its preparation history. (**B**) Schematic of training (dashed teal) and readout (solid black) cycles. After training, the state of each hysteron is recorded; this state is compared with the state after each readout cycle (i.e., at each purple dot), and the fractional difference is recorded as *d*. (**C**) An example of *d* versus the readout strain, γ_readout_, for a system of independent hysterons trained at γ_1_ = 8.

We denote the two possible states of a hysteron by (+) and (−). Its current state is determined by its previous state (that is, its history) and the current value of the shear. In terms of the energy landscape, this is equivalent to having a double-well potential such as the one shown in Fig. 1A. Any given hysteron is fully described by its flipping strains γ^+^ and γ^−^. For a system with a broad distribution of hysteron parameters, one can train the system as one would a jammed packing, with shear cycles of type (0 ➔ + γ ➔ 0 ➔ −γ ➔ 0) as in (*2*, *5*, *19*) and as shown by the teal (dashed) sawtooth curve in Fig. 1B. The readout protocol is illustrated by the black (solid) sawtooth curve, which, starting at small amplitude, has an increasing amplitude for each subsequent cycle. The amplitude of the largest previously applied strain can be read out by measuring *d*, the fraction of hysterons that have changed their state at the end of each readout cycle (shown by the purple circles on the sawtooth curve). There is a sharp cusp in *d* when the readout amplitude equals the training amplitude, as shown in Fig. 1C.

The Preisach model gives rise to a special type of memory called return-point memory, where the system remembers extremal values of the applied shear (*20*). Return-point memory has the property that memories can be stored only in a particular order; an applied strain will be erased as soon as another larger strain is applied, whereas if a smaller strain is applied, then the previously encoded memories of larger strains remain. The Preisach model and return-point memory in general have been applied to a variety of jammed systems, from simulations of binary mixtures of interacting particles (*2*) to experiments of sheared two-dimensional (2D) amorphous solids (*21*). This simple model captures many aspects of jammed systems; in particular, it produces cusp-like memories at the amplitude where the training was applied. However, it fails to describe the complexity, both in the training and in the periodic orbits themselves.

The number of driving cycles τ that a physical system takes to reach a periodic orbit is an important parameter. For small shear amplitudes, τ can be just a few cycles. As the shear amplitude grows, τ becomes large and appears to diverge at some critical amplitude (*5*, *22*). In the Preisach model, however, τ ≤ 1; this behavior can be understood by noting that each individual (independent) hysteron requires, at most, one cycle before it reaches a periodic orbit. This represents a substantial discrepancy with simulations of jammed packings.

A second important aspect of the memory formation is the period *T* of the orbit relative to the driving cycle. Simulations of frictional grains have shown that it is possible to fall into orbits that take many driving cycles for the particles to return to their original positions (*4*). For frictionless jammed packings, the period *T* grows for systems near the jamming transition (*5*). In the Preisach model, *T* = 1, which can again be understood by considering a single hysteron under cyclic shear.

### Including interactions between hysterons

Work on hysteron-based models has primarily focused on how cyclically sheared jammed systems can result in return-point memory behavior. Here, we present a generalization of the Preisach model that includes interactions between hysterons. We will show how these interactions can explain the observed existence of τ > 1 and *T* > 1, behavior that was not possible in the noninteracting model. Moreover, we show that interactions produce a form of memory that allows the recall of training amplitudes that are smaller than those subsequently applied. This memory is not stored locally in the output (that is, not in a cusp appearing at the training strain as seen, for example, in Fig. 1C) but in the overall amplitude of the response.

We introduce interactions between hysterons by making the flipping strains of hysteron *i* depend on the orientations of its *n* interacting neighbors. Since each neighbor could be in the (−) or the (+) orientation, there are 2*^n^* possible microstates, each of which induces a different value for the top (and likewise the bottom) flipping strain of hysteron *i*. How each microstate determines the flipping strains of hysteron *i* can be chosen in a variety of ways. For simplicity, we report here the choice that each microstate produces an uncorrelated random shift to the noninteracting value of the flipping strain

$$\gamma_{i}^{+}=\gamma_{i,0}^{+}+\Delta_{i,\{m\}}^{+}$$

where $\gamma_{i,0}^{+}$ is the value of the top flipping strain without interactions and $\Delta_{i,\{m\}}^{+}$is the shift to that value due to the particular microstate {*m*} of its neighbors. Similar rules were chosen for determining $\gamma_{i}^{-}$. Other possible rules, such as summing over pairwise interactions, lead to results qualitatively similar to those that we report here.

We note that this model is distinct from spin models, where hysteresis is an emergent behavior (*23*, *24*). Not only does this model allow the number of hysterons and distributions of $\gamma_{i,0}^{+}$ and $\gamma_{i,0}^{-}$ to be separately controlled but it also allows the interaction parameters (strengths $\Delta_{i,\{m\}}^{+}$ and $\Delta_{i,\{m\}}^{-}$, locality, and number of interacting hysterons) to be varied independent of the amount of hysteresis. As we shall see, this last attribute allows a perturbative analysis of how interactions enter, starting with small system size and infinitesimal interaction strength.

To implement this model, we must choose the total number *N* of hysterons in the system and the distributions of $\left[\gamma_{i}^{+},\gamma_{i}^{-},\Delta_{i,\{m\}}^{+},\;\Delta_{i,\{m\}}^{-}\right]$. We also need to specify the rules that indicate which hysterons interact with one another. We have investigated two cases: (i) a mean-field model, in which all *N* hysterons interact; and (ii) a 1D model, in which we order the *N* hysterons on a line (with periodic boundary conditions) and add interactions between each hysteron and its *L* neighbors on either side. We measured memory effects in the 1D model for large systems *N* > > *L* and found them to be indistinguishable in both shape and magnitude from our results for the mean-field simulations as long as each hysteron interacts with the same number of neighbors (i.e., the size of the mean-field system, *N*_MF_, is chosen to be the same as 2*L* + 1 in the 1D model).

To determine the initial state of the system, a random sequence of *N* (−) and (+) states is chosen, and the system is “relaxed” so that any unstable hysterons are stabilized. During a shear cycle, the hysterons are flipped one at a time, and the system is relaxed after each step. For each configuration, τ and *T* are recorded.

We follow standard training and readout protocols (*2*, *5*). We apply one or more training cycles and record the state of each hysteron; this is our reference state. To read out, we apply cycles of increasing amplitude and record the fractional difference *d* between the current state and the reference state after each readout cycle (shown as purple circles in the readout curve of Fig. 1B). A typical plot of *d* versus γ_readout_ for a noninteracting system of hysterons is shown in Fig. 1C.

To explore the memory capacity of this model, we train systems with a protocol involving two amplitudes: First, we fully train the system at a shear γ_1_ (that is, apply as many training cycles as needed so that the system is in a periodic state at that amplitude of shear), then we apply a single cycle at a second shear amplitude γ_2_. In a noninteracting model, if γ_2_ > γ_1_, then the second amplitude would immediately (i.e., within a single cycle) erase any memory of the first input; in that case, two copies of the same system trained at different values of γ_1_ but with the same value of the larger amplitude γ_2_ would look identical. When interactions are added, a single larger-amplitude shear cycle does not necessarily erase the memory of the smaller, previously stored, input amplitude. Thus, it is possible to observe more complex behavior in the storage of multiple memories in this model.

### Results from simulations

To determine the role of interactions, we simulate the model for different values of *N* and with distributions of $\left[\gamma_{i,0}^{+},\gamma_{i,0}^{-},\Delta_{i,\{m\}}^{+},\;\Delta_{i,\{m\}}^{-}\right]$ as described in Materials and Methods below. The qualitative behavior of the model does not depend critically on the choices made for these distributions as long as the distributions are sufficiently broad.

**Fig. 2:**
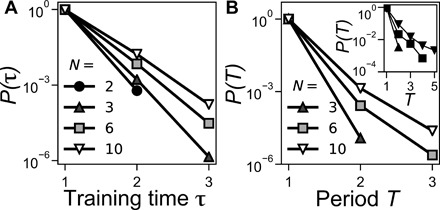
Probability of τ and *T*. Probability distributions of (**A**) τ and (**B**) *T* in a mean-field model of interacting hysterons where the parameter *A* in the distribution of interaction strengths, defined in Materials and Methods, was chosen to be *A* = 0.5. Inset in (B) shows *P*(*T*) versus *T* from simulations of cyclically sheared jammed packings, adapted from (*5*). In the inset, higher probability curves correspond to lower pressure where the system is closer to the jamming threshold.

We find that interactions between hysterons allow for both τ and *T* values greater than 1, a result consistent with what has been seen in spin systems with emergent hysteresis (*23*, *24*). Figure 2 (A and B) shows the probabilities *P*(τ) and *P*(*T*) for finding a configuration with a training time τ or period *T* for different system sizes in the mean-field model. In both cases, the probability decays approximately exponentially with τ or *T* and increases as the number of interacting hysterons is increased. It is possible to get τ = 2 for systems as small as *N* = 2 interacting hysterons and to get *T* = 2 for systems as small as *N* = 3.

The inset of Fig. 2B shows *P*(*T*) versus *T* for simulations of jammed systems cyclically sheared in the quasi-static limit (*5*). In those simulations, *P*(*T*) also decayed approximately exponentially with *T* and increased as the system was brought closer to the jamming transition where the range of elastic interactions increases. This is consistent with our mean-field model where *P*(*T > 1*) increases with the number of interacting neighbors *N*.

### Memories of multiple training inputs

As emphasized above, noninteracting hysterons give rise to a hierarchy of memories so that when a larger-amplitude training shear is applied, it erases all memories of previous training with smaller amplitudes. When we introduce interactions, this is no longer the case; for τ > 1, a single shear cycle is, by definition, not sufficient to bring the system to a periodic state. It is therefore possible that when a larger strain is applied to a system that has already been trained at a smaller amplitude, there will be a signature left of the initial trained state. Such behavior was investigated in three systems showing “multiple-transient memories”: charge-density waves (*25*, *26*), non-Brownian suspensions (*10*–*12*), and the park-bench model (*1*, *16*). In those cases, there is a memory of the smaller-amplitude behavior that eventually vanishes as more training occurs at the larger amplitudes. The memory appears as a cusp in the readout at both training amplitudes. As training continues, the cusp at the smaller amplitude disappears, leaving only the single memory associated with the larger-amplitude input.

**Fig. 3:**
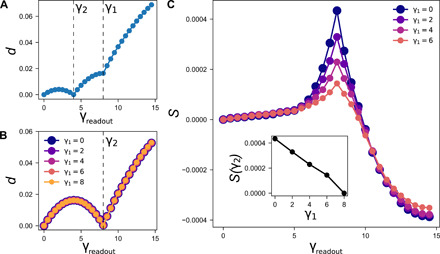
Readout for interacting model. (**A**) Averaged readout curve for *N* = 11 mean-field configurations with γ_1_ > γ_2_ in an interacting system with interaction strength *A* = 0.1. The curve, which shows two sharp cusps at γ_1_ and γ_2_, is nearly indistinguishable from that produced by noninteracting systems trained in the same way. However, *d*(γ_1_) is precisely zero for noninteracting systems and is small but nonzero for interacting systems. (**B**) Averaged readout curve for *N* = 11 mean-field configurations with different values of γ_1_, all less than γ_2_. All systems were trained at γ_2_ = 8 and have interaction strength *A* = 0.1. Dashed line shows γ_2_. The data points (shown with different colors and symbols sizes for clarity) lie very nearly on top of one another, so it is difficult to see the difference between them. (**C**) The same data as shown in (B) but with the curve for *d*_γ1 = γ2 = 8_ subtracted off, leaving *S* ≡ *d* − *d*_γ1 = γ2_. This reveals that there is extra structure in the readout indicating that a memory of the initial (smaller amplitude) input is still encoded in the system. Inset shows the magnitude of *S* at γ_2_ as a function of γ_1_; this magnitude is roughly linear in γ_1_.

We have investigated the possibility of a second memory in our model of interacting hysterons. When we apply our two-amplitude training protocol with a second training pulse that has a smaller amplitude than the first one, γ_2_ < γ_1_, we see behavior that is similar to the noninteracting case of the Preisach model but with some differences. This is shown in Fig. 3A. As in the Preisach model, there is a sharp cusp at both γ_1_ and γ_2_. However, when interactions are introduced, the cusp at γ_1_ no longer occurs at *d* = 0, appearing instead at a finite value *d* > 0.

When the second training pulse has a larger amplitude than the first one, γ_2_ > γ_1_, the system retains a memory of the smaller amplitude input at γ_1_. This would not have been possible in the noninteracting case. Figure 3B shows the readout for systems trained at different values of γ_1_ and the same value of γ_2_ (γ_2_ = 8) with γ_1_ < γ_2_. The curves are nearly the same but not identical; there is a signal that is buried in the small differences between the curves. This signal can be uncovered by subtracting off the “background” (the curve *d*_γ2 = γ1_ corresponding to γ_1_ = γ_2_ = 8, which is equivalent to training fully at γ_2_): *S* ≡ *d* − *d*_γ2 = γ1_. As shown in Fig. 3C, *S* not only has a cusp at γ_2_, but the separate curves have a magnitude at γ_2_ that is a roughly linear function of γ_1_ as shown in the inset to Fig. 3C. The value of γ_1_ can be determined by the magnitude of *S*(γ_2_).

**Fig. 4:**
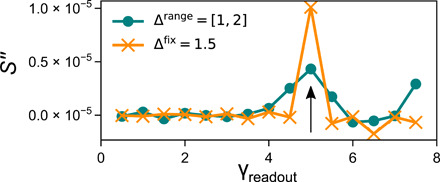
*S* and *S″* for simplified interactions. *S″*, the second derivative of the background-subtracted readout, versus γ_readout_ for *N = 2* systems with γ_1_ = 0 and γ_2_ = 8. For (Δ^fix^) interactions, there is a peak sharply localized around γ_readout_ = 5, indicated by the black arrow. For (Δ^range^) interactions, the peak is centered on the same value but is broadened. The (Δ^range^) data points also show a peak just below γ_readout_ = 8, corresponding to cases where flipping strains happened to have the same sign so that the resulting “splitting” was smaller than 1.

When we fix the interactions to be either (+∆^fix^) or (−∆^fix^) (so that the splitting between values of $\gamma_{i}^{+}$ is either 0 or 2∆^fix^, depending on the state of its neighbors), we find that the readout, *S*, has a second cusp structure that appears at (γ_2_ − 2∆^fix^). This appears as a peak in *S″*, the second derivative of the readout curve with respect to γ_readout_, as shown in Fig. 4. Adding a distribution, ∆^range^, around ∆^fix^ shows a corresponding broadening of the cusp around (γ_2_ − 2∆^fix^), also seen in Fig. 4. *S″* thus contains more than just the average interaction strength: For small interaction strength, *S″* provides the distribution of interactions strengths between the hysterons.

We note that this is different from the double cusps that were seen in the case of multiple transient memories in suspensions and charge-density waves (*10*–*12*, *25*, *26*). In that case, the two cusps were determined by the two training amplitudes; here, only one cusp is related to the amplitude γ_2_; the other is determined by the interaction strength |∆^fix^|.

### Analysis of two- and three-hysteron systems

Focusing on small (*N* = 2 and 3) systems provides insight into the periodicity and memory capacity of interacting hysterons. It has proven useful to describe such systems as directed graphs that represent each microstate of the system as a node with arrows showing how the system flows to other nodes as shear is applied (*19*, *27*). Here, we use directed graphs to illustrate which states are visited and in what order, under cyclic shear of a particular amplitude. For small *N*, this makes it possible to enumerate all possible cases with a particular periodicity or training time. In such diagrams, we impose the rule that only one hysteron can flip at a time.

**Fig. 5:**
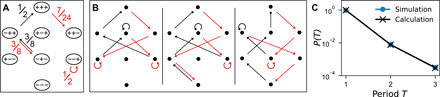
Contributions to *T* = 2 for three-hysteron systems. (**A**) Template for *N* = 3 directed graphs showing all possible microstates and the probability of each transition for a set of three interacting hysterons under cyclic shear in the convenient case of simplified hysteron parameters as described in case (ii) of Materials and Methods. Note that the probabilities are the same for all arrows from one given horizontal level to another [for example, an arrow from (−++) to (−−+) has the same probability as the one from (+−+) to (+−−)] and are symmetric under inversion of the entire system [for example, an arrow from (−++) to (+++) has the same probability as the one from (+−−) to (−−−)]. (**B**) The three *N* = 3 directed graphs (up to hysteron swap, time reversal, and + ➔ − inversion) with *T* = 2. The probability of finding *T* = 2 is calculated by finding the probabilities and multiplicities of each graph. Note that each arrow contributes an additional factor as specified in (A), so that cycles with more arrows (for example, *T* > 1 compared with *T* = 1) tend to be less likely. (**C**) Comparison of calculation and simulation for simplified hysteron parameters as described in case (ii) of Materials and Methods.

Although analytic calculations quickly become intractable for large system sizes, an analysis of the case *N* = 3 is possible and illustrates the nature of the approximately exponential decay of *P*(*T*) with *T* shown in Fig. 2B. Figure 5B shows all the directed graphs for *N* = 3 with *T* = 2 (up to hysteron swap, time reversal, and (+) ➔ (−) inversion, which contribute to the corresponding multiplicities). The probability of any such graph will depend on the multiplicity of each diagram and the probability of each arrow connecting two nodes. Although multiplicity of a given loop increases for larger numbers of microstates visited, there is also a fractional factor associated with each outgoing arrow (shown in Fig. 5A for a particular choice of parameters), so that higher period loops are suppressed by some small fraction to a high power. This leads to the generic exponential decrease of *P*(*T*) with *T*. We performed *T* = 2 and 3 calculations for *N* = 3 with simplified model parameters and found excellent agreement with simulation results as shown in Fig. 5C.

**Fig. 6:**
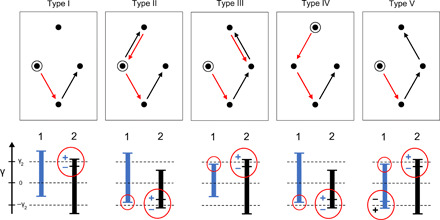
Contributions to τ = 2 for two-hysteron systems. The five *N* = 2 directed graphs that give rise to τ = 2 behavior and hence contribute to the background-subtracted readout *S* when γ_1_ = 0. The starting configuration is circled in black; arrows represent changes in microstate due to shear cycles at γ_2_. Underneath each directed graph is the associated bar diagram showing hysterons 1 and 2. The training strain γ_2_, 0, and −γ_2_ are shown as dashed lines. When a single hysteron has multiple values of its top or bottom flipping strain, the state of the other hysteron corresponding to each value is shown. Limiting constraints (that is, flipping strains that must be within ~Δ of γ_2_ or −γ_2_) are circled; note that type I has only one such constraint, while types II to IV have two and type V has three.

Analysis of systems with small interaction strength allows us to think of the interactions as perturbations about the noninteracting case and shows the origin of the second memory. This can be seen most clearly in two-hysteron systems, *N* = 2. For this case, Fig. 6 shows all possible configurations with τ = 2, which are the only systems that contribute to the background-subtracted readout *S* for γ_1_ = 0. Each of these configurations has at least one flipping strain that straddles ±γ_2_ due to an interaction. This is a limiting constraint (represented by a red circle in the figure) as it requires a bar end to be within ~Δ of γ_2_ or −γ_2_. Type I configurations require only one limiting constraint, while the others require two or more. (The bar ends that are not circled must lie roughly within the intervals indicated between the three horizontal dashed lines but do not have be within ~Δ of γ_2_ or −γ_2_.) Therefore, when Δ < < |γ_2_|, type I configurations dominate because their contribution scales with ∆ rather than with ∆^2^ or ∆^3^. In the limit of small ∆, we can therefore analyze the effect of interactions by studying only this one case.

For γ_1_ between 0 and γ_2_, an additional configuration type becomes relevant. This is a more complex case in which τ = 1 yet different amplitude cycles lead to different outcomes; however, the same general picture applies. For any value of γ_1_ < γ_2_, these diagrams can be used to demonstrate that there is no structure at γ_readout_ = γ_1_ in *S*. This memory is therefore in a different class from those found in suspensions with multiple transient memories.

**Fig. 7:**
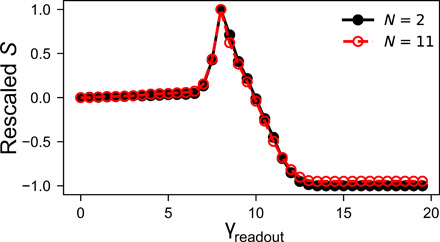
*S* for two different system sizes. Comparison of background-subtracted readout S for *N* = 2 and *N* = 11 systems with interaction strength *A* = 0.05. The curves agree very well. Both curves are for γ_1_ = 0 and γ_2_ = 8 and are rescaled to equal 1 at γ_2_.

As long as the interaction strength is small, increasing *N* does not result in qualitatively different behavior, as shown in Fig. 7. For small ∆, we can thus understand even large systems as a perturbation around the type I behavior of two-hysteron interactions. For larger ∆, we must include higher-order diagrams.

## DISCUSSION

The probabilities of getting *T* > 1 and τ > 1 and the exponential decay of *P*(*T*) with *T*, as observed in (*5*), are successes of this model. In cyclically sheared jammed packings, we expect systems closer to jamming (that is, at lower pressure) to have longer-range interactions, effectively coupling a larger number of rearranging regions. The increase in *P*(*T* > 1) with system size in our systems of hysterons is therefore consistent with the general trend seen in (*5*), where *P*(*T* > 1) increases with decreasing pressure. The effect of relative location of the interacting regions has been studied in the paper by Keim and Paulsen (*28*).

We see two distinct types of memory in Fig. 3C. As in noninteracting systems of hysterons, γ_2_ is stored locally in the location of the cusp of the readout signal. There is no local signature such as a cusp in the response associated with the amplitude of γ_1_ if γ_1_ < γ_2_ in accord with simulations of jammed systems (*6*). This is in contrast to the cusp-like second memory that was found in non-Brownian suspensions and charge-density waves (*10*–*12*, *25*, *26*). However, there is still a memory of γ_1_ that is embedded in the magnitude of the readout; by calibrating the response, it is possible to determine the exact value of γ_1_. This is a previously unidentified form of memory. Our results suggest that we can reinterpret readout curves from simulations of jammed systems for different numbers of training cycles (*2*) likewise as a memory of the number of training cycles applied to the system.

It is especially exciting that this memory provides a way to measure the strength of the interactions present in an interacting system. We showed that for two Dirac delta functions at (±∆^fix^), we can read out the interaction strength directly from the readout signal. This survives to broader distributions. Thus, the shape of the readout signal *S* provides information about typical interaction strengths in the system. This could provide a new, possibly unique, method to determine this crucial parameter in real systems. A natural next step is to apply this training protocol to simulations of a jammed system. If our interacting model successfully captures the memory behavior of such systems, it could provide a simple method for measuring the distribution of interaction strengths between rearrangements.

Systems of hysterons have been used successfully to reproduce many elements of cyclically sheared jammed packings. We have reported here further similarities for small systems when interactions are included. However, this is very unexpected, since systems of hysterons differ fundamentally from jammed packings; after all, there is a vast gap in the number of degrees of freedom between these two systems, and it is far from obvious that binary variables can adequately describe real, continuous systems. It may be that hysteron models describe well the behavior of jammed systems near their periodic orbit but fail to capture early transient behavior of large systems. Further simulations and experiments are essential for understanding when and why hysterons successfully capture the essence of their much more complex jammed counterparts. Nevertheless, the model that we have introduced here is of sufficient generality that it can be applied to a wide variety of systems, including those with more discrete variables than jammed packings.

## MATERIALS AND METHODS

For simulations of the mean-field model, we report the results for three types of situations: (i) The interaction strengths $\Delta_{i,\{m\}}^{+}$ and $\Delta_{i,\{m\}}^{-}$ for hysteron *i* are chosen uniformly from $\left[-A\;(\gamma_{i,0}^{+}-\gamma_{i,0}^{-}),\;+A(\gamma_{i,0}^{+}-\gamma_{i,0}^{-})\right]$ so that the interactions are scaled by the length of the hysteron they affect. That is, longer hysterons are more likely to have their flipping strains shifted by larger values. We choose *A* between 0 and 0.5. The distribution of initial hysteron lengths $\left(\gamma_{i,0}^{+}-\gamma_{i,0}^{-}\right)$ and midpoints $(\gamma_{i,0}^{+}+\gamma_{i,0}^{-})/2$ are drawn randomly from between [0,20] and [−25,25], respectively. (ii) The distribution of initial hysteron lengths $\left(\gamma_{i,0}^{+}-\gamma_{i,0}^{-}\right)$ and midpoints $(\gamma_{i,0}^{+}+\gamma_{i,0}^{-})/2$ are drawn randomly from between [9.99,10.01] and [−0.01,0.01], respectively, so that all hysterons are initially essentially identical. The interaction strengths $\Delta_{i,\{m\}}^{+}$ and $\Delta_{i,\{m\}}^{-}$ for hysteron *i* are chosen uniformly from $\left[-A\;(\gamma_{i,0}^{+}-\gamma_{i,0}^{-}),\;+A(\gamma_{i,0}^{+}-\gamma_{i,0}^{-})\right]$ with *A* = 0.1. (iii) We fix the values of $\Delta_{i,\{m\}}^{+}$ to be (±∆^fix^) so that $\gamma_{i}^{+}=\gamma_{i,0}^{+}\pm \Delta^{\text{fix}}$ and likewise for $\gamma_{i}^{-}$. In this case, the shifts are not scaled by the length of the hysteron that they affect. We choose the distribution of $\left(\gamma_{i,0}^{+}-\gamma_{i,0}^{-}\right)$ and $(\gamma_{i,0}^{+}+\gamma_{i,0}^{-})/2$ to be drawn randomly from between [5,20] and [−25,25], respectively. In all cases, the results are averaged over between 10^4^ and ~10^7^ independent configurations.
